# Simultaneous bilateral total knee arthroplasty in patients with end-stage hemophilic arthropathy: a mean follow-up of 6 years

**DOI:** 10.1038/s41598-018-19852-7

**Published:** 2018-01-25

**Authors:** Chao Jiang, Yan Zhao, Bin Feng, Jiliang Zhai, Yanyan Bian, Guixing Qiu, Xisheng Weng

**Affiliations:** Department of Orthopedics, Peking Union Medical College Hospital, Chinese Academy of Medical Sciences & Peking Union Medical College, No. 1 Shuaifuyuan Wangfujing, Dongcheng District, Beijing, 100730 China

## Abstract

To investigate the safety, cost-effectiveness, and clinical outcomes of simultaneous bilateral total knee arthroplasty (TKA) in hemophilic arthropathy (HA), the requirements for transfusions, complications, costs, hospital stays, Hospital for Special Surgery (HSS) knee scores, knee range of motion (ROM) and revision rates were compared between simultaneous bilateral and unilateral TKA in HA patients. A total of 36 patients and 54 knees were included. Compared to the unilateral group, the bilateral group did not require more transfusions (2.39 ± 3.13 vs 0.83 ± 1.38 units of RBCs, p > 0.05) or consumption of coagulation factors (50091.67 ± 25168.5 vs 46477.78 ± 11348.32 IU, p > 0.05), complications rate (13/36 vs 6/18, p > 0.05), hospital stay (32.39 ± 19.77 vs 29.11 ± 12.67 days, p > 0.05), or costs excluding prostheses (14945.41 ± 6634.35 vs 14742.12 ± 5746.78 US dollars, p > 0.05). Additionally, the two groups exhibited similar medium-term knee HSS scores (83.67 ± 7.11 vs 81.00 ± 10.35, p > 0.05) and ROM (89.39° ± 13.66° vs 88.91° ± 12.90°, p > 0.05). Our data indicate that bilateral TKA is a safe and cost-effective treatment for HA with similar medium-term results compared to unilateral TKA.

## Introduction

**Figure 1:**
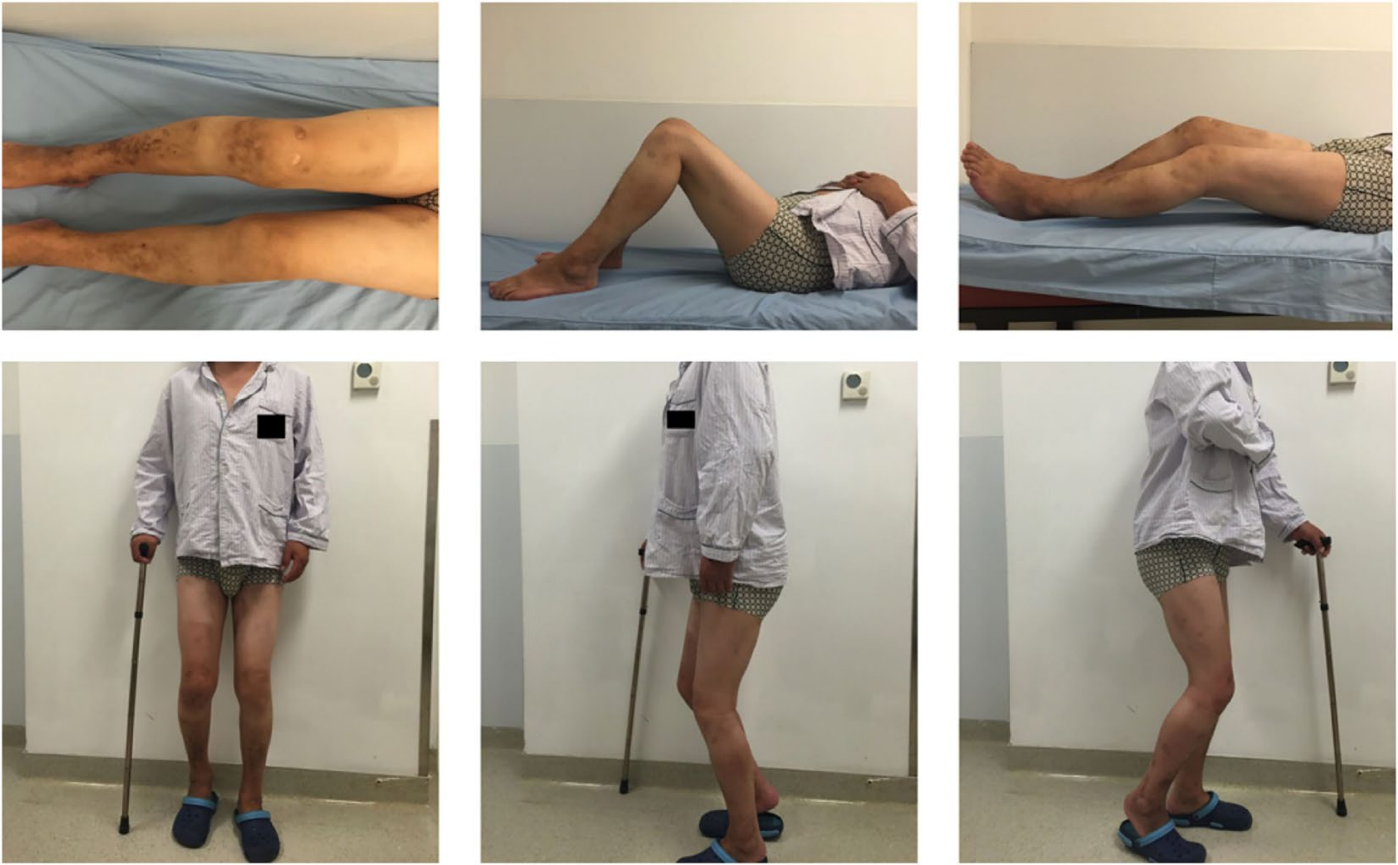
Preoperative images of a patient included in the study. The range of motion was approximately 40 degrees (extension) to 90 degrees (flexion) on the right side and 15 to 90 degrees on the left side. Flexion contracture was observed in both knee joints.

Hemophilic arthropathy (HA) is a common and occasionally inevitable complication that affects greater than 90% of patients with hemophilia before the age of 30^1^. Multiple joints, including the knee and hip, are often involved, leading to loss of function and permanent disabilities in end stage^2–5^ (Fig. 1). The pathogenesis of HA begins with hemophilic synovitis induced by recurrent hemarthrosis, followed by joint erosion with cartilage damage and erosion of adjacent bones^6^. The most important approach to prevent HA involves eliminating intra-articular hemorrhage by regular factor replacement therapy (FRT)^7^. However, 70–80% of patients with hemophilia can not receive appropriate treatment and are thus at increased risk of developing HA^8^, and the knee joint is regarded as one of the most vulnerable joints^9^.

Total knee arthroplasty (TKA) has been considered an optimal choice for treatment of HA^10–16^, and FRT is imperative in maintaining an adequate level of clotting factors to minimize blood loss perioperatively^8,17,18^. Given that HA of both knees is often involved, bilateral TKA is always unavoidable in the end stage. As staged bilateral TKA requires repeated clotting factor infusions that may induce the development of inhibitory against coagulation factors^19,20^ as well as increases in hospitalization costs, simultaneous bilateral TKA may be considered a better treatment option. We conducted this retrospective study of 36 patients with a mean follow-up of 6 years to investigate the safety, cost-effectiveness, and medium- and long-term clinical outcomes of patients with end-stage HA receiving simultaneous bilateral TKA compared to unilateral TKA. We propose a hypothesis that the clinical results of simultaneous bilateral TKA were not inferior to unilateral TKA in HA patients.

## Methods

### Study design

**Figure 2:**
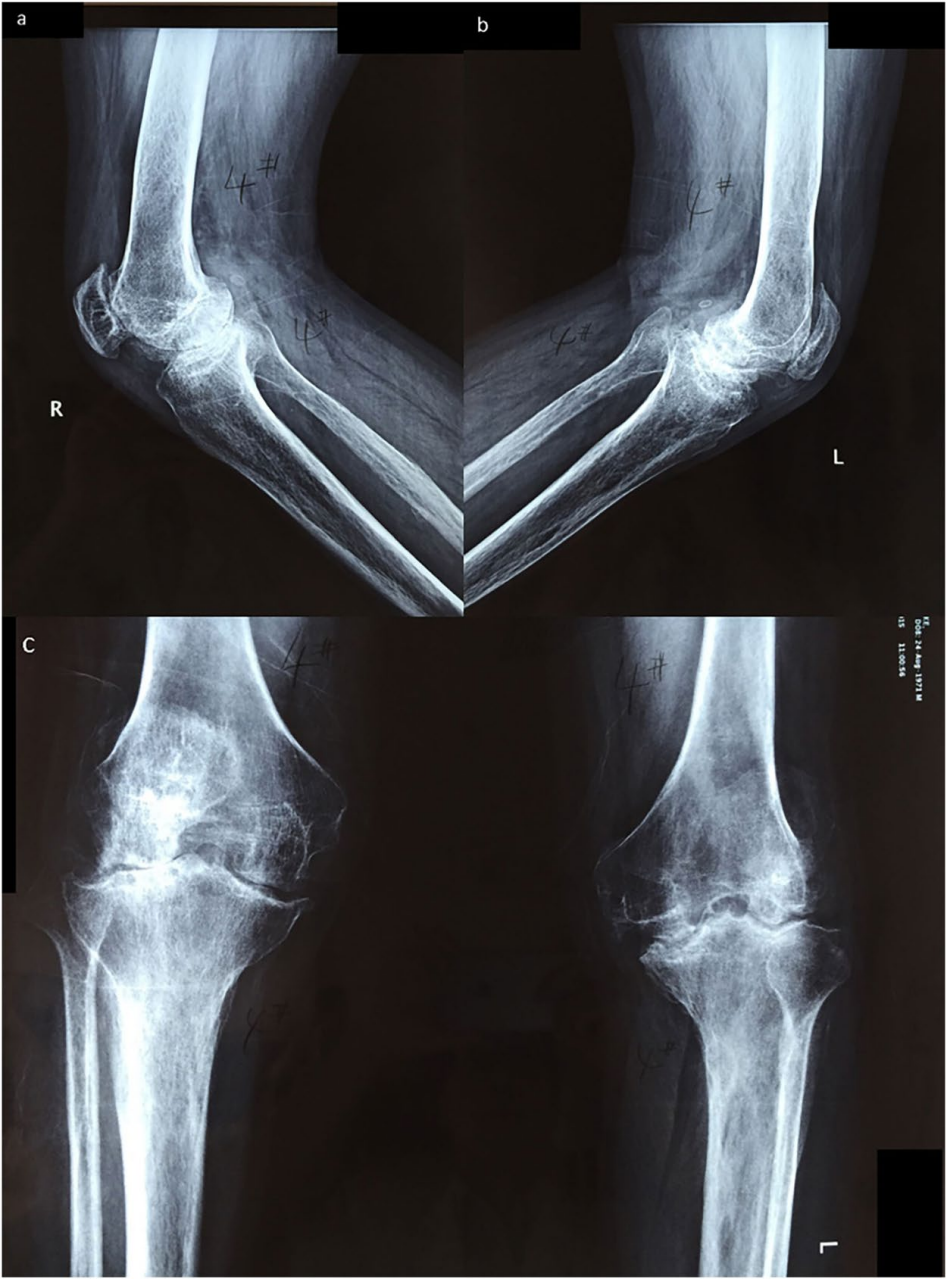
Preoperative X-ray image of the same patient presented in Fig. 1. The film indicated end-stage hemophilic arthropathy in both knee joints. (**a**) Lateral view of the right knee joint. (**b**) Lateral view of the left knee joint. (**c**) Anteroposterior view of both knee joints. Patient information is masked to protect the patient’s privacy.

**Figure 3:**
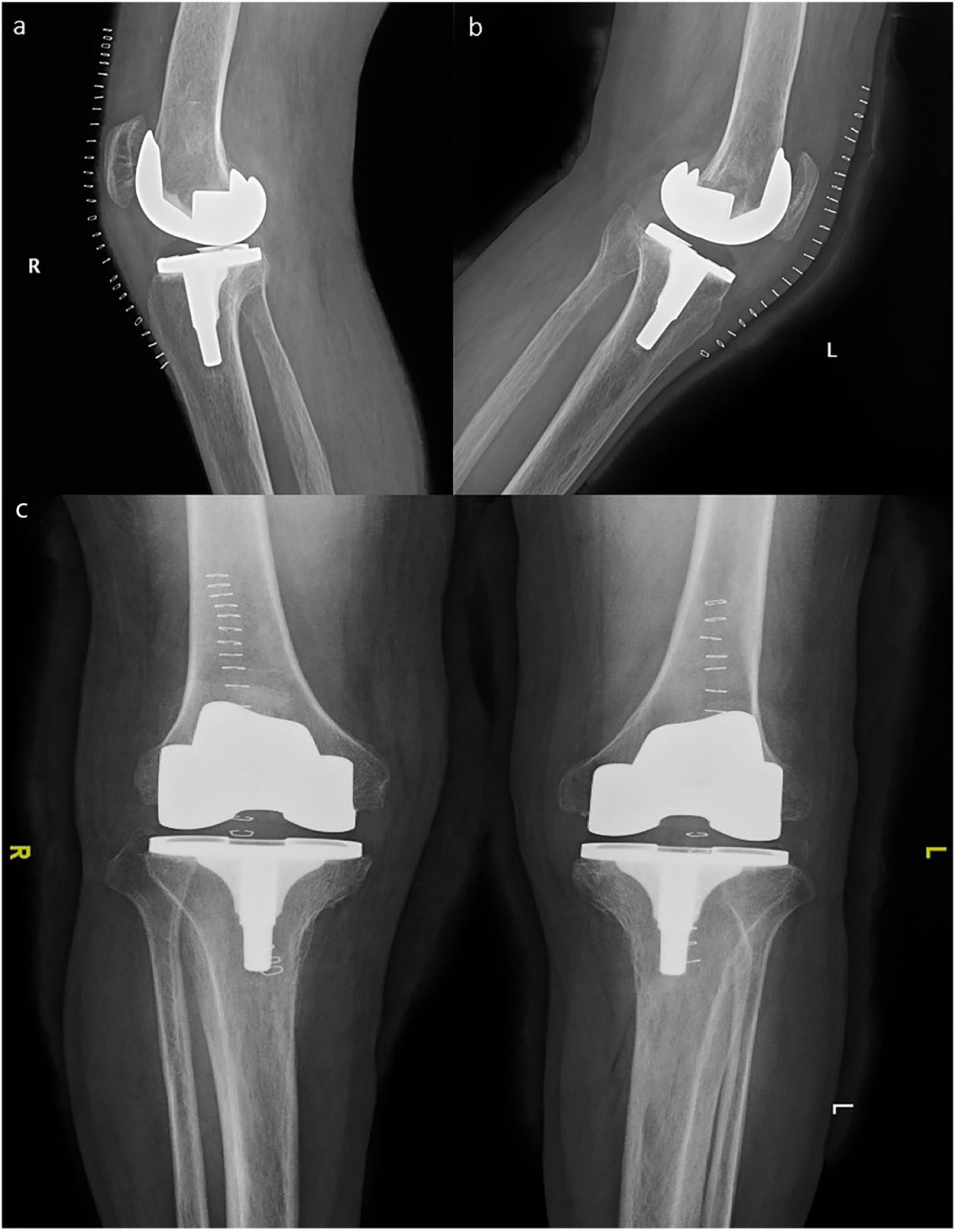
Postoperative X-ray image of the same patient presented in Fig. 1. (**a**) Lateral view of the right knee joint. (**b**) Lateral view of the left knee joint. (**c**) Lnteroposterior view of both knee joints.

We searched the database of a single center for patients older than 18 years with end-stage HA (Fig. 2) who were treated with TKA from April 2005 to April 2015. The inclusion criteria were as follows: a) patients with hemophilia who had incapacitating HA; b) only TKA was performed during one admission. Patients were excluded if a) the records were incomplete, or b) they underwent other surgical procedures, including total hip arthroplasty (THA), ankle joint arthroplasty or elbow joint arthroplasty during the same hospitalization. A total of 37 patients were identified. One patient underwent one TKA procedure at first admission followed by simultaneous TKA and THA 3 years later. Therefore, this case was excluded from the study. Of the remaining 36 patients, 18 patients underwent unilateral TKA, and the other 18 underwent simultaneous bilateral TKA. No other staged bilateral TKA cases were noted. Patients who received bilateral TKA under single anesthesia by the same surgeon were included in the “simultaneous bilateral” group. No patients receiving staged bilateral TKAs with separate anesthesia during one hospitalization were included in our study (Fig. 3).

All subjects were unaware of the purpose of the study. After explanation of the nature and possible consequences of the study, written informed consent was obtained from each patient for print and online open-access publication of their outcomes and related data. The patient in Fig. 1 agreed via written informed consent to publication of the images shown. The Institutional Review Board of Peking Union Medical College Hospital approved this study, and all methods were performed in accordance with guidelines (Declaration of Helsinki) for biomedical research. All materials, data and associated protocols in this study are available to readers and the publishing team.

### Surgical procedure and perioperative management

One surgical team with the same chief surgeon (the corresponding author, W) in this hospital performed all procedures with the patients under general anesthesia, following a previously described protocol^11^. The tourniquet, same type of implant and intramedullary guides were used in all cases with the midline parapatellar approach. In cases with simultaneous bilateral TKA, the second leg (the side with the worse deformity) was sterilized and subjected to the operation after completion of the skin incision and dressing of the first leg. Patients received prophylactic antibiotics once 30 minutes before skin incision, and one extra dose was administered within 24 hours after surgery. Tranexamic acid (TXA, 1000 mg) was intravenously administered to all patients 15 minutes before skin incision, and topical TXA solution (1000 mg) was applied intra-articularly to each operated knee approximately 5 minutes before closure. As hemophilic patients tend to bleed, no anti-thrombotic therapy was used in all cases routinely. Drainage were removed 48 to 72 hours after surgery.

The injection of coagulation factor VIII (or prothrombincomplex for type B hemophilia) started half an hour before the operation by bolus infusion and was repeated during the perioperative period. The concentrations of blood coagulation factors were typically maintained at approximately 100% on the day of the surgery, approximately 80% during the first 3 postoperative days and approximately 60% during the fourth to sixth postoperative days. The dose was adjusted based on the target mentioned above, and 50 IU/kg was typically used as the initial dose; thereafter, the concentrations of coagulation factors were gradually decreased until discharge, as mentioned in our previous study^22^. All serum clotting factor levels were monitored daily during the first week postoperatively and once every other day thereafter until discharge. Blood transfusion was performed in patients who exhibited hemoglobin levels less than 7 g/dL or less than 10 g/dL with ischemic symptoms. Patients were discharged upon wound healing (removal of staples); resolution of fever, symptoms of superficial infection, and active articular hemorrhage; and when they could walk longer than 100 meters without any passive assistance and the total range of motion (ROM) showed significant improvement (postoperative total ROM no less than 90 degree if achievable). Thus, the patients in our study had a quite a long hospital stay.

### Data collection

Demographic profiles, preoperative lab assays (complete blood count, coagulation function and coagulation factor activity, factor consumption), and details of the surgery and hospitalization (time of operation, blood loss and transfusion during surgery, transfusion after surgery, hemoglobin level after surgery, occurrence of complications, length and costs of hospitalization, prosthesis infection, and revision rates) were summarized and compared between the groups. Hospital for Special Surgery (HSS) knee scores (total score of 115) and knee ROM were recorded before surgery, at discharge and at the latest follow-up.

### Statistical analysis

Data were analyzed with SPSS (IBM Corp. Released 2013. IBM SPSS Statistics for Windows, Version 22.0. Armonk, NY: IBM Corp). Failure was defined as removal of prosthesis for any reason. Student’s t-test was used for comparison of continuous variables with a normal distribution. A non-parametric test was performed for comparison of continuous variables with a non-normal distribution. The chi-square test or Fisher’s exact test was used to compare categorical variables. A log-rank test was used to compare the survival curve of the two groups. A two-sided p-value of <0.05 was considered significant.

## Results

### Demographic features

Thirty-six patients were included in this study. Eighteen patients underwent unilateral TKA, and the remaining 18 patients underwent simultaneous bilateral TKA. No significant differences in age, weight, type of hemophilia and comorbidities were noted between the two groups. The mean follow-up time was 72.61 months (range 23–144 months) for the bilateral TKA group and 75.94 months (37–138 months) for the unilateral TKA group. Preoperative lab result analysis revealed no significant differences in preoperative hemoglobin (Hb), prothrombin time (PT), activated partial thromboplastin time (APTT) and coagulation factor activity between the two groups **(**Table 1**)**.

**Table 1:** Demographic profiles and preoperative lab assays of patients. Unilateral and bilateral groups refer to patients who received unilateral TKA and simultaneous bilateral TKA, respectively. Age is presented as the mean ± SD with the range, and weight is presented as the mean ± SD.

	Unilateral (n = 18)	Bilateral (n = 18)	p-value
Age (years)	30.06 ± 11.31 (15–61)	35.33 ± 11.42 (18–56)	>0.05
Weight (kg)	71.39 ± 13.32	68.11 ± 12.85	>0.05
Type of hemophilia (A/B)	16/2	17/1	>0.05
Comorbidities^*^	3/18	4/18	>0.05
Mild hypertension	2	3	>0.05
Diabetes mellitus	1	1	>0.05
Red blood cells (×10^12^/L)	5.17 ± 0.51	4.96 ± 0.39	>0.05
Hemoglobin (g/L)	143.78 ± 11.12	144.53 ± 20.05	>0.05
Platelets (×10^9^/L)	217.17 ± 62.93	212.47 ± 58.96	>0.05
White blood cells (×109/L)	5.73 ± 1.06	5.6 ± 1.38	>0.05
C reactive protein (mg/L)	4.33 ± 6.12	7.39 ± 11.99	>0.05
Activated partial thromboplastin time (s)	80.92 ± 20.25	85.13 ± 25.38	>0.05
Prothrombin time (s)	12.06 ± 0.78	11.77 ± 0.82	>0.05
Coagulation factor activity (%)	4.7 ± 7.56	2.83 ± 2.58	>0.05
Follow-up period (months)	75.94 ± 42.62	72.61 ± 34.75	>0.05

*Comorbidities include hypertension and diabetes mellitus.

### Operative details and perioperative outcomes

Significant differences in operation time (156.67 ± 60.17 vs 261.11 ± 58.5 minutes, p < 0.01) and postoperative hemoglobin levels were noted between the two groups but did not result in increased transfusion requirements perioperatively (2.39 ± 3.13 vs 0.83 ± 1.38 units of RBCs, p > 0.05). The cost of hospitalization, excluding prostheses (14945.41 ± 6634.35 vs 14742.12 ± 5746.78 US dollars, p > 0.05), the consumption of coagulation factors (50,091.67 ± 25,168.5 vs 46,477.78 ± 11,348.32 IU, p > 0.05), and the length of hospitalization (32.39 ± 19.77 vs 29.11 ± 12.67, p > 0.05) did not differ significantly between the two groups (Table 2).

**Table 2:** Operation and hospitalization details. Data are presented as the mean ± SD. Blood loss refers to the blood lost during the operation. Transfusion during and after the operation refer to red blood cells only, and one unit of red blood cells was acquired from 200 mL of blood from healthy donors. The postoperative hemoglobin level represents the lowest hemoglobin level after surgery. Complications included wound infection, abnormal healing of the wound, possible nerve impairment, intra-articular hemorrhage, and adverse gastrointestinal (GI) reactions.

	Unilateral (n = 18)	Bilateral (n = 18)	p-value
Operation time (min)	156.67 ± 60.17	261.11 ± 58.5	<0.01
Blood loss (mL)	133.89 ± 111.57	258.33 ± 100.89	<0.01
Transfusion during operation (U)	0.78 ± 1.56	0.11 ± 0.47	>0.05
Postoperative drainage (mL)	599.44 ± 527.95	763.33 ± 470.96	<0.01
Postoperative transfusion (U)	0.83 ± 1.38	2.39 ± 3.13	>0.05
Postoperative hemoglobin level (g/L)	99.33 ± 17.57	75.61 ± 13.81	<0.05
Coagulation factor consumed (IU)	46477.78 ± 11348.32	50091.67 ± 25168.5	>0.05
Length of hospitalization (days)	29.11 ± 12.67	32.39 ± 19.77	>0.05
Cost of hospitalization (US dollars)	20831.42 ± 7849.48	26968.69 ± 5628.93	<0.01
Cost of hospitalization (prostheses excluded, US dollars)	14742.12 ± 5746.78	14945.41 ± 6634.35	>0.05

Postoperative complications included blisters, superficial wound infection, nerve impairments, venous thrombus embolism (VTE), cardiac events, intra-articular hemorrhage and other related events. Based on our observations, 15 of 36 patients with prostheses in the bilateral TKA group and 7 of 18 in the unilateral TKA group experienced different types of perioperative complications. Symptomatic deep-vein thrombosis (DVT), pulmonary embolism (PE) or cardiac events did not occur in either group. Two patients in the bilateral TKA group and 1 in the unilateral TKA group suffered from intra-articular hemorrhage after surgery. Among these patients, 1 from the bilateral group required surgical removal of the hematoma. No 30-day mortality occurred in our study. In total, no significant differences in perioperative complications were noted between the two groups (Table 3).

**Table 3:** Details of postoperative complications. Patients with more than one complication were considered multiple times according to the quantities of complications. Nerve impairment included pain, paralysis and hypoesthesia. Adverse GI reactions included nausea, emesis, and abdominal pain.

	Unilateral	Bilateral	p-value
Blisters	1/18	3/36	>0.05
Superficial wound infection	1/18	1/36	>0.05
Nerve impairment	3/18	7/36	>0.05
Intra-articular hemorrhage	1/18	2/36	>0.05
Revision	1/18	2/36	>0.05
Deep infection	1/18	1/36	>0.05
Aseptic loosening	0	1/36	>0.05
Total complications	7/18	15/36	>0.05

### Prosthesis failure and revisions

During the mean follow-up period of 6 years (range 13–144 months), 1 patient in each group developed deep infection (5.56% vs 2.78%, p > 0.05); furthermore, 1 patient in the bilateral group (left side in bilateral TKA) experienced aseptic prosthetic loosening. These 3 patients underwent revision surgeries (5.56% vs 5.56%, p = 0.937). At the latest follow-up, the prosthesis survival rate was 94.4% (17/18) in the unilateral group, 94.4% (34/36) in the bilateral group and 94.4% (51/54) overall.

**Figure 4:**
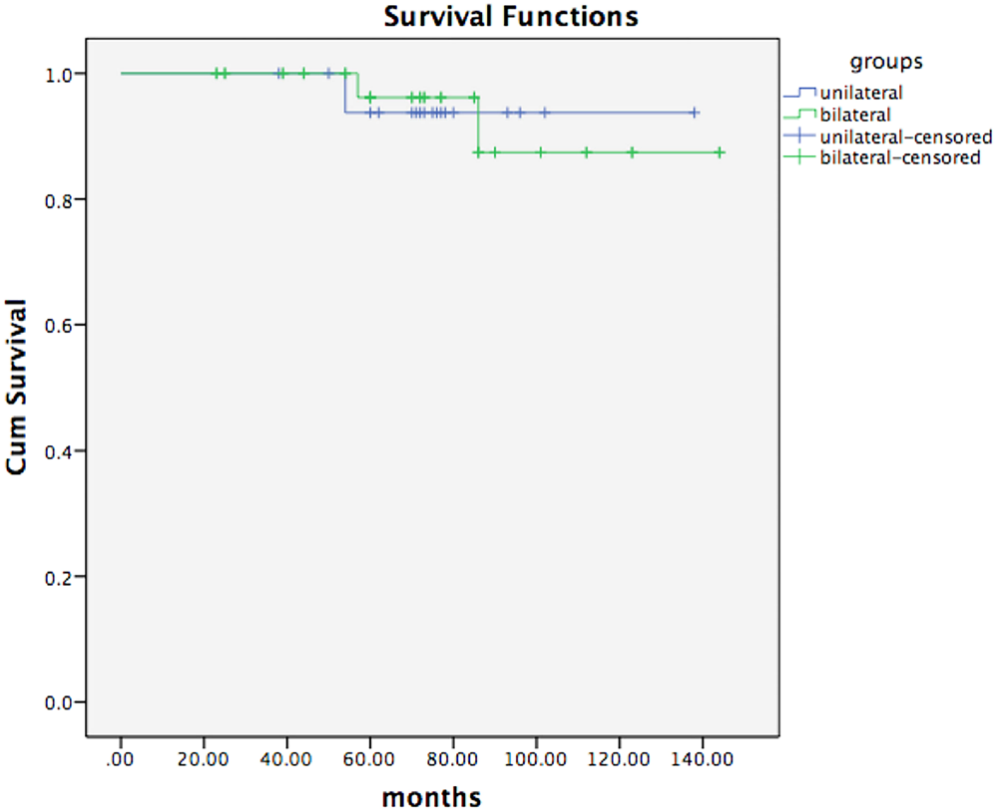
Survival curves of both groups.

Survival curves are presented in Fig. 4, at p = 0.996 according to the logrank test.

### HSS and ROM

The HSS knee score and ROM data were available for all 36 patients. The two groups exhibited similar HSS scores (53.50 ± 13.99 vs 47.44 ± 20.22, p > 0.05), extension (16.33 ± 14.81 vs 20.83 ± 17.26, p > 0.05), flexion (84.86 ± 30.69 vs 84.72 ± 34.83, p > 0.05) and total ROM (67.92 ± 27.13 vs 63.89 ± 33.10, p > 0.05) preoperatively. Significant improvements in HSS score and ROM were noted in both groups after TKA and at the latest follow-up. No significant differences were observed between the two groups at the latest follow-up for HSS score (83.67 ± 7.11 vs 81.00 ± 10.35, p > 0.05), extension (2.53 ± 2.88 vs 3.06 ± 5.18, p > 0.05), flexion (91.92 ± 12.61 vs 92.17 ± 15.27, p > 0.05) or total ROM (89.39 ± 13.66 vs 88.91 ± 12.90, p > 0.05) (Table 4).

**Table 4:** Details of HSS (full score of 115), knee extension, flexion and total ROM before surgery, at discharge and at the latest follow-up.

	Unilateral (n = 18)	Bilateral (n = 36)	p-value
Preop HSS	47.45 ± 20.22	53.50 ± 13.99	>0.05
Discharge HSS	73.89 ± 13.14	76.50 ± 9.78	>0.05
Latest follow-up HSS	81.00 ± 10.35	83.67 ± 7.11	>0.05
Preop extension	20.83 ± 17.26	16.33 ± 14.81	>0.05
Preop flexion	84.72 ± 34.83	84.86 ± 30.69	>0.05
Preop ROM	63.89 ± 13.10	67.92 ± 27.13	>0.05
Discharge extension	2.78 ± 5.21	1.95 ± 3.44	>0.05
Discharge flexion	91.95 ± 16.01	89.06 ± 13.69	>0.05
Discharge ROM	89.17 ± 14.68	87.11 ± 14.30	>0.05
Latest follow-up extension	3.06 ± 5.18	2.53 ± 2.88	>0.05
Latest follow-up flexion	92.17 ± 15.27	91.92 ± 12.61	>0.05
Latest follow-up ROM	88.91 ± 12.90	89.39 ± 13.66	>0.05

## Discussion

This study was a retrospective study of a consecutive group of cases, and we confirmed good clinical results for simultaneous bilateral TKA in patients with HA. Considering the possible advantages of simultaneous bilateral TKA, as mentioned above, all patients with severe HA in both knees were advised to have simultaneous bilateral TKA at our center. Only one patient underwent staged bilateral TKA during our study period, and the second TKA was simultaneously performed with a THA on the same side; this case was excluded from our study.

Complications often occur in patients who receive TKA. In our study, despite the increased operation time and perioperative bleeding and reduced hemoglobin levels observed after surgery, patients who underwent simultaneous bilateral TKA did not require increased transfusion or experience increased perioperative complications, hospital costs (prostheses excluded), or hospital stays. Indeed, the extra cost of simultaneous bilateral TKA was attributed to the prosthesis, which is an inevitable expense, regardless of the procedure used. In our country, the operation fee is determined by the surgical procedure and not by the operation time; thus, increased operation time costs were not observed.

Although it remains controversial whether simultaneous bilateral TKA in osteoarthritis (OA) should be recommended in bilateral OA patients, our results with properly selected patients were promising. In HA patients, repeated injection of coagulation factor is not only expensive but also associated with the risk of the development of factor inhibitors, which is a critical condition in hemophiliac patients. Therefore, simultaneous bilateral TKA appears to be more urgent in HA than in OA. Patients with hemophilia are at increased risk of significant bleeding during perioperative periods; therefore, FRT is crucial for optimizing the coagulation function. However, FRT may result in extra costs and the production of auto-antibodies^8^. By avoiding two-staged procedures, simultaneous bilateral TKA is a potential solution to this dilemma. Indeed, as we predicted, simultaneous bilateral TKA did not require increased consumption of coagulation factors and can even reduce total consumption compared to staged TKA. Overall, bilateral TKA is more affordable and minimizes the possibility of inducing inhibitory antibodies against coagulation factors.

Previous studies have reported no difference in improvement of joint function between simultaneous and staged bilateral or unilateral TKA^21,23,25^. Our results were consistent with these studies. No significant differences in HSS score and ROM were noted before surgery between the groups in our study, whereas both HSS score and ROM showed significant improvement after TKA in both groups, and this improvement persisted to the latest follow-up in most patients. Because both groups had similar ROM and HSS scores at the latest follow-up, simultaneous bilateral TKA resulted in similar knee function reconstruction and pain relief at the medium-term follow-up. We believe that simultaneous bilateral TKA would benefit postoperative rehabilitation because restrictions from the other leg would be eliminated. However, this should be investigated in further studies.

In previous studies, the five- and ten-year prosthetic survival rates were approximately 90% for TKA surgery in HA patients^24,25^, which is similar to the results of our study. We experienced a total of 3 revisions in the mean follow-up of 6 years among 54 prostheses, for a revision rate of 5.6%. Among these 3 cases, one patient from the bilateral TKA group was diagnosed with aseptic prosthetic loosening at 9 years postoperatively, which might have been due to aggravating HA of the nearby hip causing improper postoperative lower limb alignment. In addition, one deep infection occurred in each group, which were diagnosed at 24 and 17 months postoperatively in the bilateral and unilateral TKA groups, respectively. In these two cases, *Streptococcus anginosus* was detected in the patient who underwent bilateral TKA, and coagulase-negative *Staphylococcus* was found in the unilateral TKA case. Revision surgery was performed after 8 weeks of antibiotic therapy in both cases. However, due to the limited number of cases included in our study, we could not perform statistical analysis of prosthesis survival rates.

Our study has several limitations. Ideally, we should compare staged bilateral TKA with simultaneous bilateral TKA. However, only one patient with bilateral HA of the knees underwent staged TKA during our study, as simultaneous bilateral TKA was most often chosen. Second, given that this disease is uncommon, only a small number of patients were included in our study. Thus, certain parameters, such as the prosthesis survival rate, could not be assessed with the limited number of patients. Finally, our results are only applicable for a 6-year follow-up; further longer-term results are needed.

In spite of the advantages of simultaneous bilateral TKA in patients with hemophilia that has been depicted in a number of publications as well as the results of ourselves study, such treatment is still supposed to be debatable according to a recent report^2^. In addition, both a systemic review and a meta-analysis showed that more complications are witnessed in patients without hemophilia who underwent bilateral TKA than those who underwent unilateral TKA^26,27^. Therefore, more persuasive data concerning TKA in patients with hemophilia are still awaited^2^.

## Conclusion

Our data appear to indicate that bilateral TKA in hemophilia patients is a safe and cost-effective treatment for HA that offers medium-term results comparable to those of unilateral TKA. Further large-scale studies are required to obtain a definitive conclusion.

### Data availability statement

All the data and related protocols in this article are available to Editorial Board Members and every reader.
